# Comparative analysis of complete mitochondrial genome sequences confirms independent origins of plant-parasitic nematodes

**DOI:** 10.1186/1471-2148-13-12

**Published:** 2013-01-18

**Authors:** Tahera Sultana, Jiyeon Kim, Sang-Hwa Lee, Hyerim Han, Sanghee Kim, Gi-Sik Min, Steven A Nadler, Joogn-Ki Park

**Affiliations:** 1 Department of Biological Sciences, Inha University, Incheon, 402-751, Republic of Korea; 2 Graduate Program in Cell Biology and Genetics, College of Medicine, Chungbuk National University, Cheongju, 361-763, Republic of Korea; 3 Division of Forest Insect Pests and Diseases, Korea Forest Research Institute, Seoul, 130-012, Republic of Korea; 4 Korea Polar Research Institute, Songdo Techno Park, Yeonsu-gu, Incheon, 406-840, Republic of Korea; 5 Department of Entomology and Nematology, University of California, Davis, CA, 95616, USA; 6 Graduate Program in Cell Biology and Genetics and Department of Parasitology, College of Medicine, Chungbuk National University, Cheongju, 361-763, Republic of Korea

**Keywords:** Plant parasitism, Tylenchomorpha, Chromadorea, Nematoda, Mitochondrial genome, Molecular phylogeny

## Abstract

**Background:**

The nematode infraorder Tylenchomorpha (Class Chromadorea) includes plant parasites that are of agricultural and economic importance, as well as insect-associates and fungal feeding species. Among tylenchomorph plant parasites, members of the superfamily Tylenchoidea, such as root-knot nematodes, have great impact on agriculture. Of the five superfamilies within Tylenchomorpha, one (Aphelenchoidea) includes mainly fungal-feeding species, but also some damaging plant pathogens, including certain *Bursaphelenchus* spp. The evolutionary relationships of tylenchoid and aphelenchoid nematodes have been disputed based on classical morphological features and molecular data. For example, similarities in the structure of the stomatostylet suggested a common evolutionary origin. In contrast, phylogenetic hypotheses based on nuclear SSU ribosomal DNA sequences have revealed paraphyly of Aphelenchoidea, with, for example, fungal-feeding *Aphelenchus* spp. within Tylenchomorpha, but *Bursaphelenchus* and *Aphelenchoides* spp. more closely related to infraorder Panagrolaimomorpha. We investigated phylogenetic relationships of plant-parasitic tylenchoid and aphelenchoid species in the context of other chromadorean nematodes based on comparative analysis of complete mitochondrial genome data, including two newly sequenced genomes from *Bursaphelenchus xylophilus* (Aphelenchoidea) and *Pratylenchus vulnus* (Tylenchoidea).

**Results:**

The complete mitochondrial genomes of *B. xylophilus* and *P. vulnus* are 14,778 bp and 21,656 bp, respectively, and identical to all other chromadorean nematode mtDNAs in that they contain 36 genes (lacking *atp8*) encoded in the same direction. Their mitochondrial protein-coding genes are biased toward use of amino acids encoded by T-rich codons, resulting in high A+T richness. Phylogenetic analyses of both nucleotide and amino acid sequence datasets using maximum likelihood and Bayesian methods did not support *B. xylophilus* as most closely related to Tylenchomorpha (Tylenchoidea). Instead, *B. xylophilus,* was nested within a strongly supported clade consisting of species from infraorders Rhabditomorpha, Panagrolaimomorpha, Diplogasteromorpha, and Ascaridomorpha. The clade containing sampled Tylenchoidea (*P. vulnus, H. glycines*, and *R. similis*) was sister to all analyzed chromadoreans. Comparison of gene arrangement data was also consistent with the phylogenetic relationships as inferred from sequence data. Alternative tree topologies depicting a monophyletic grouping of *B. xylophilus* (Aphelenchoidea) plus Tylenchoidea, Tylenchoidea plus Diplogasteromorpha (*Pristionchus pacificus*), or *B. xylophilus* plus Diplogasteromorpha were significantly worse interpretations of the mtDNA data.

**Conclusions:**

Phylogenetic trees inferred from nucleotide and amino acid sequences of mtDNA coding genes are in agreement that *B. xylophilus* (the single representative of Aphelenchoidea) is not closely related to Tylenchoidea, indicating that these two groups of plant parasites do not share an exclusive most recent common ancestor, and that certain morphological similarities between these stylet-bearing nematodes must result from convergent evolution. In addition, the exceptionally large mtDNA genome size of *P. vulnus*, which is the largest among chromadorean nematode mtDNAs sequenced to date, results from lengthy repeated segments in non-coding regions.

## Background

Nematodes are among the most common, abundant and ecologically diverse animal groups. Free-living species inhabit almost every environment, and are extremely abundant in soils and aquatic sediments, both freshwater and marine [1,2]. Most nematode diversity is represented by these free-living species [3]. Nematode parasites of plants and animals are also frequently encountered, and cause reductions in agricultural productivity, and disease in humans, domestic animals and wildlife [4]. Plant-parasitic nematodes attack a wide variety of commercial crops, mainly causing damage to root tissues [5] that impacts on the physiology of the host plant [6], particularly water transport. Plant parasites are usually microscopic, and may feed on plant tissues as ectoparasites or endoparasites, depending on the species. Among the most remarkable specializations of plant parasites are sedentary endoparasites that induce nurse cells in host plant roots that serve as metabolic sinks and nematode feeding sites, sustaining the sedentary female nematode during its lifetime in the host tissue. Other plant-parasitic species have more diverse feeding habits. For example, the pine wilt nematode *Bursaphelenchus xylophilus*, which is transmitted by wood-boring beetles, has both phytophagous and mycophagous phases in its life history.

Molecular phylogenies based on SSU rDNA [7-11] indicate that three traditional orders of plant parasites, Dorylaimida Pearse 1942, Triplonchida Cobb 1920, and Tylenchida Thorne 1949 evolved independently. Modern taxonomic systems for nematodes [12,13] are mainly based on the phylogenetic framework provided by SSU rDNA in combination with new interpretations of developmental and morphological features. In the taxonomy of De Ley and Blaxter [12,13], which is used herein, orders Dorylaimida and Triplonchida (within class Enoplea) are retained, whereas the phylogenetic framework guides taxonomic reorganization of a diverse assemblage of species (free-living and parasitic) within suborder Tylenchina (order Rhabditida, class Chromadorea). Within Tylenchina, the infraorder Tylenchomorpha includes stylet-bearing nematodes with representatives that are plant-parasites, insect associates or parasites (e.g., Sphaerulariidae), and fungal feeders. Several of the superfamilies within Tylenchomorpha include important plant pathogens [3,14], particularly Tylenchoidea (which includes root-knot and lesion nematodes, among others), but also Aphelenchoidea, the superfamily that includes insect associates, fungivores, and plant-associates that in some cases are either direct causative agents of plant disease (*B. xylophilus*), or through fungal feeding are associated with root disease (e.g., *Aphelenchus avenae*). The relationship and taxonomy of groups now classified as Tylenchomorpha, and specifically the relationship of aphelenchs to other stylet-bearing Tylenchina, has been a topic of debate among nematode taxonomists for several decades.

Some arguments for a close relationship between aphelenchs and Tylenchomorpha have been based on morphology, for example, the highly similar protrusible stylets (stomatostylets) in these taxa [15], or aspects of female genital structure [16]. However, Siddiqi [14] suggested that the similar stylets of these nematodes arose independently, with the stylet of aphelenchs derived from diplogasteromorph ancestors, thus rejecting the concept of their close evolutionary relationship. Siddiqi [14] recognized aphelenchs as a separate evolutionary group [17-19].

In recent years, phylogenetic relationships inferred from SSU rDNA sequences [7-11] have provided an alternative framework for nematode classification. Representatives of Tylenchomorpha including certain aphelenchs (Aphelenchidae) comprised a clade, with the fungivorous Aphelenchidae typically the sister group to plant-parasitic tylenchomorphs. However, the Aphelenchoidea were not monophyletic [9-11], with plant pathogens such as *Aphelenchoides* spp. and *Bursaphelenchus* spp. (Aphelenchoididae) either unresolved among Tylenchina [11] or more closely related to Panagrolaimomorpha, but in the latter case with support levels varying substantially, depending on inference method [9,10]. Recent fine structure reconstructions of the pharynx also call into question the monophyly of Aphelenchoidea, revealing lack of cellular homology underlying structures previously assumed to unite Aphelenchidae and Aphelenchoididae [20]. It is unclear if artefacts such as base compositional bias or long-branch-attraction may be influencing the position of *Bursaphelenchus* spp. and other Aphelenchoididae in SSU trees, but this potential caveat has been suggested as one possible explanation for non-monophyly [12]. For this reason, independent phylogenetic evidence from other gene loci is needed to test this and other hypotheses of nematode relationships.

As in many other metazoan groups, comparative analysis of nematode mitochondrial (mt) genome information (nucleotide and amino acid sequences, and gene arrangement) appears useful for resolving relationships at different divergence levels [21-24]. To date, 63 complete mt genomes have been reported for nematodes (52 chromadoreans and 11 enopleans), however, taxon sampling is biased toward parasites of vertebrates. Despite their importance to agriculture, Tylenchomorpha are undersampled for complete mitochondrial genomes. Published reports include the complete sequence of the burrowing nematode [25]*Radopholus similis* (Tylenchoidea, Pratylenchidae), and partial genome, but complete coding sequences of the soybean cyst nematode [26]*Heterodera glycines* (Tylenchoidea, Hoplolaimidae). However, the focus of these studies has been on idiosyncratic features of the mt sequences and not phylogenetic context. In this study, we determined the complete mitochondrial DNA sequences of *Bursaphelenchus xylophilus,* the first representative of the Aphelenchoidea and *Pratylenchus vulnus*, the second representative of the family Pratylenchidae, respectively, and used these data for inferring phylogenetic relationships among the major groups of chromadoreans.

## Results and discussion

### Gene contents and organization

**Figure 1:**
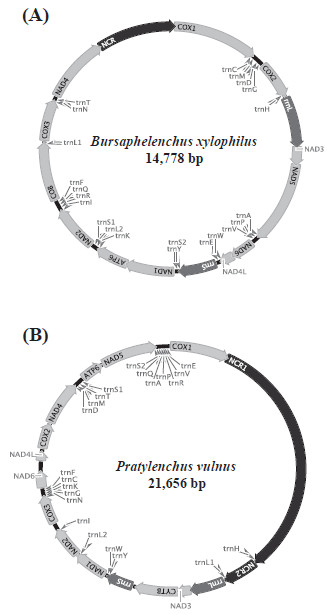
**Circular gene maps of the complete mitochondrial genome for *Bursaphelenchus xylophilus *(A) and *Pratylenchus vulnus *** (**B**). All genes are encoded in the same direction and 22 tRNA genes are designated by a single-letter abbreviation. The two leucine and two serine tRNA genes are labeled, according to their anticodon sequence, as L1 (*trnL-uag*), L2 (*trnL-uaa*), S1 (*trnS-ucu*), and S2 (*trnS-uga*), respectively.

The complete mitochondrial genomes of *B. xylophilus* (GenBank accession number: GQ332424) and *P. vulnus* (GenBank accession number GQ332425) are 14,778 bp and 21,656 bp, respectively. The mtDNA of *P. vulnus* is the largest among chromadorean nematode mtDNAs published to date. It is much larger than *Radopholus similis* (16,791 bp), the first complete mtDNA for Tylenchoidea [25], and *P. vulnus* is third largest among all nematodes reported to date, following two Mermithida, *Romanomermis culicivorax* (26,194 bp) and *Hexamermis agrotis* (24,606 bp). Unlike enopleans, the mtDNA genomes of chromadorean nematodes do not normally exceed 17 kb. The exception is the soybean cyst nematode *Heterodera glycines* that is estimated to have a 21–22 kb-circular mtDNA chromosome [26]. The remarkably large size of *P. vulnus* mtDNA is due to abnormally lengthy non-coding regions that harbor tandemly repeated sequences. This feature was also reported for the root-knot nematode *Meloidogyne javanica* (Tylenchoidea), which contains a 7 kb control region with different numbers of tandemly repeated sequence units [27]. In some Enoplea (mermithids), size variation ranging from 19 to 34 kb is relatively common and is attributed to a ‘hypervariable’ segment that includes both coding and putative nonfunctional regions (see [28] for more details). The complete mitochondrial genomes of *B. xylophilus* (Figure 1A) and *P. vulnus* (Figure 1B) contain 36 genes that comprise 12 protein-coding genes (PCGs) but lacking *atp8*, 22 tRNA and 2 rRNA genes, all encoded in the same direction. This gene content is common to all other nematode mtDNAs so far sequenced, except for *Trichinella spiralis* (Enoplea) in which *atp8* is also encoded [29]. The details of gene order and size for mtDNA of *B. xylophilus* and *P. vulnus* are shown in Table 1. The nucleotide composition of the mtDNA genome of each species has a strong bias toward A+T-richness with overall content of 83.5% in *B. xylophilus* (51.7% T, 31.8% A, 11.1% G, and 5.4% C) and 73.9% in *P. vulnus* (42.1% T, 31.8% A, 16.3% G, and 9.8% C), respectively (Table 2). The A+T richness in these two species is related to the propensity for high frequency use of T-rich and/or A-rich codons in their protein-coding genes and higher A+T content in non-coding regions (see following section).

**Table 1:** **The mitochondrial genome organization of *Bursaphelenchus xylophilus *(Bx) and *Pratylenchus vulnus *(Pv)**

***Bursaphelenchus xylophilus***					***Pratylenchus vulnus***				
**Gene**	**Positions and lengths of nucleotide sequences**	**No. of nt (bp)**	**Initiation and termination codons**	**Intergenic sequence**	**Gene**	**Positions and lengths of nucleotide sequences**	**No. of nt (bp)**	**Initiation and termination codons**	**Intergenic sequence**
*cox1*	1-1563	1563	ATT/TAA	11	*cox1*	1-1533	1533	ATA/TAA	7*
*trnC*	1575-1628	54		5	*NCR1*	1534-8380	6847		
*trnM*	1634-1687	54		20	*trnH*	8381-8434	54		
*trnD*	1708-1762	55		3	*NCR2*	8435-9335	901		
*trnG*	1766-1819	54			*trnL1*	9336-9393	58		2
*cox2*	1820-2509	690	ATT/TAG	−2	*rrnL*	9396-10290	895		21
*trnH*	2508-2562	55			*nad3*	10312-10641	330	ATT/TAG	9
*rrnL*	2563-3510	948			*cob*	10651-11757	1107	ATA/TAG	123
*nad3*	3511-3840	330	ATT/TAA	1	*rrnS*	11881-12566	686		
*nad5*	3842-5410	1569	ATT/TAA	−1	*trnY*	12567-12620	54		
*trnA*	5410-5464	55			*trnW*	12621-12676	56		
*trnP*	5465-5521	57			*nad1*	12677-13549	873	TTG/TAA	1
*trnV*	5522-5578	57			*trnL2*	13551-13606	56		1
*nad6*	5579-6013	435	ATT/TAA	−1	*nad2*	13608-14429	822	ATA/TAG	
*nad4L*	6013-6246	234	ATA/TAA		*trnI*	14430-14485	56		187
*trnW*	6247-6301	55			*cox3*	14673-15449	777	ATA/TAG	
*trnE*	6302-6356	55			*trnN*	15450-15501	52		
*rrnS*	6357-7056	700			*trnG*	15502-15557	56		1
*trnS2*	7057-7111	55			*trnK*	15559-15615	57		4
*trnY*	7112-7167	56			*trnC*	15620-15673	54		
*nad1*	7168-8040	873	ATT/TAA	−5	*trnF*	15674-15728	55		
*atp6*	8036-8630	595	ATT/T		*nad6*	15729-16157	429	TTG/TAG	326
*trnK*	8631-8690	60			*nad4L*	16484-16714	231	ATT/TAG	118
*trnL2*	8691-8745	55			*cox2*	16833-17513	681	ATA/TAG	67
*trnS1*	8746-8798	53			*nad4*	17581-18792	1212	ATA/TAA	9
*nad2*	8799-9623	825	ATT/TAA	11	*trnD*	18802-18857	56		
*trnI*	9635-9694	60		5	*trnM*	18858-18911	54		42
*trnR*	9700-9752	53		5	*trnT*	18954-19007	54		45
*trnQ*	9758-9811	54			*trnS1*	19053-19111	59		11
*trnF*	9812-9865	54			*atp6*	19123-19704	582	ATT/TAG	75
*cob*	9866-10967	1102	ATA/T		*nad5*	19780-21240	1461	ATT/TAA	
*trnL1*	10968-11022	55			*trnS2*	21241-21293	53		10
*cox3*	11023-11790	768	ATT/TAA		*trnQ*	21304-21358	55		2
*trnN*	11791-11844	54			*trnA*	21361-21416	56		7
*trnT*	11845-11898	54			*trnP*	21424-21476	53		
*nad4*	11899-13128	1230	ATA/TAA		*trnR*	21477-21530	54		
*NCR*	13129-14778	1650			*trnV*	21531-21586	56		1
					*trnE*	21588-21645	58		

*7 bp intergenic sequence between *trnE* and *cox1.*

**Table 2:** **Nucleotide composition of *Bursaphelenchus xylophilus *(Bx) and *Pratylenchus vulnus *(Pv)**

**Nucleotide**	**Length (bp)**		**A (%)**		**C (%)**		**T (%)**		**G (%)**		**A+T (%)**	
	**Bx**	**Pv**	**Bx**	**Pv**	**Bx**	**Pv**	**Bx**	**Pv**	**Bx**	**Pv**	**Bx**	**Pv**
Entire sequence	14778	21656	31.8	31.8	5.4	9.8	51.7	42.1	11.1	16.3	83.5	73.9
Protein coding sequence	10182	10002	27.1	26.7	6.3	8.7	53.4	48.4	13.2	16.2	80.5	75.1
Codon position												
1st	3394	3334	28.5	27.6	6.6	9.0	46.8	43.0	18.1	20.4	75.3	70.6
2nd	3394	3334	19.2	18.3	10.4	12.5	55.7	51.8	14.7	17.4	74.9	70.1
3rd	3394	3334	33.5	34.4	1.9	4.4	57.7	50.3	6.8	10.9	91.2	84.7
Ribosomal RNA gene sequence	1648	1581	39.1	30.2	5.0	11.5	46.2	39.2	9.7	19.1	85.3	69.4
Transfer RNA gene sequence	1214	1216	39.1	35.0	4.9	6.9	45.6	42.2	10.5	16.0	84.7	77.2
Non coding region	1650	7748	47.7	37.4	0.7	11.3	51.0	35.5	0.7	15.8	98.7	72.9

### Protein-coding genes (PCGs)

Twelve PCGs were identified from both species, ranging from 234 bp (*nad4L*) to 1,569 bp (*nad5*) for *B. xylophilus* and from 231 bp (*nad4L*) to 1,533 bp (*cox1*) for *P. vulnus*. Total length of the 12 PCGs of *B. xylophilus* mtDNA is 10,182 bp, accounting for 68.9% of its total mtDNA genome length. This value is slightly lower than most chromadoreans, such as *Caenorhabditis briggsae* (71.4%), *Ascaris suum* (72.8%) and *Anisakis simplex* (73.8%). In contrast, total length of the 12 PCGs of *P. vulnus* mtDNA (10,002 bp) accounts for less than half (46.2%) of its entire genome length. This lowered proportion of PCG sequence in *P. vulnus* is due to extraordinarily lengthy non-coding regions. This is very uncommon in other chromadorean nematode mtDNAs except for *Heterorhabditis bacteriophora* (56.84%), but known for enoplean mitochondrial genomes, for example, *Hexamermis agrotis* (40.2%), and *Romanomermis culicivorax* (37.9%).

Out of 12 PCGs of *B. xylophilus* mtDNA, nine (*cox1-cox3, nad1-nad3, nad5, nad6* and *atp6*) are inferred to use ATT as the start codon, whereas three (*nad4, nad4L* and *cob*) start with ATA (Table 1). Although ATG is known to be the most commonly used initiation codon for mitochondrial PCGs [30], use of others such as TTG is very common in some other nematode mtDNAs, including *Anisakis simplex* (9 of 12 genes) and *Enterobius vermicularis* (8 of 12 genes). Out of 12 *P. vulnus* PCGs, six (*cox1-cox3, nad2, nad4,* and *cob*) are inferred to use ATA as the start codon. Four genes (*atp6, nad3, nad4L,* and *nad5*) start with ATT, whereas *nad1* and *nad6* start with TTG (Table 1). The most commonly used start codon for *P. vulnus* (ATA) is also the most frequently used in *Xiphinema americanum* mtDNA (11 of 12 PCGs). More rarely, TTA is used as a start codon in nematodes, for example, *Ancylostoma doudenale* (*cox1*), *Brugia malayi* (*nad2*) and *Steinernema carpocapsae* (*nad2*, *nad4,* and *nad6*).

For *B. xylophilus*, nine genes (*cox1, cox3, nad1-nad4, nad4L, nad5* and *nad6*) terminate with TAA and *cox2* uses TAG as its termination codon; *atp6* and *cob* are inferred to terminate with incomplete stop codon T. Among these, *cox2* is inferred to overlap with *trnH* by two nucleotides (AG) and *nad1* is also inferred to overlap with *atp6* by five nucleotides. The truncated (incomplete) termination codon (terminate with ‘T’) is inferred for the *atp6* and *cob* of *B. xylophilus*; truncated termination ‘T’ is believed to be completed by polyadenylation [31]. For *P. vulnus,* eight genes (*atp6, cox2, cox3, nad2, nad3, nad4L, nad6,* and *cob*) are predicted to terminate with TAG and four (*cox1, nad1, nad4,* and *nad5*) with TAA, all without overlapping any adjacent gene boundary. The use of TAA as a termination codon in *P. vulnus* is generally consistent with another Tylenchoidea, *H. glycines*[26], but is in marked contrast to the idiosyncratic codon usage found in the other sequenced member of the same superfamily, *R. similis* in which TAA, the canonical stop codon in the standard invertebrate genetic code, is reassigned to encode Tyr [25].

As in other published nematode mtDNAs, the mitochondrial PCGs of these two species are notably biased toward using amino acids encoded by T-rich codons. The three most frequently used codons have more than two Ts in a triplet (Table 3): in *B. xylophilus*, TTT (Phe: 19.12%), TTA (Leu: 13.52%), and ATT (Ile: 7.66%); in *P. vulnus*, TTT (Phe: 14.49%), TTA (Leu: 11.31%), and ATT (Ile: 5.64%). These T-rich codons account for 40.3% (*B. xylophilus*) and 31.44% (*P. vulnus*) of the total codons used. In addition, there is nonrandom use of synonymous codons; avoidance of C in the third codon position is pronounced in two- and four-fold degenerate codon families. As an example, there are large differences in the relative frequency of codons for phenylalanine usage between TTT (19.12%) and TTC (0.53%) in *B. xylophilus* and between TTT (14.49%) and TTC (1.14%) in *P. vulnus*. The higher frequency of amino acids encoded by T-rich codons, and unequal synonymous codon usage with bias against C-rich codons is consistent with the high percentage of A+T content in the nucleotide composition of PCGs (A+T content of 80.5% and 75.1% for *B. xylophilus* and *P. vulnus,* respectively; Table 2).

**Table 3:** **Codon usage of 12 protein coding genes of *Bursaphelenchus xylophilus *(Bx) and *Pratylenchus vulnus *(Pv) mtDNAs**

**Codon**	**AA**	**No.**		**%**		**Codon**	**AA**	**No.**		**%**	
		**Bx**	**Pv**	**Bx**	**Pv**			**Bx**	**Pv**	**Bx**	**Pv**
TTT	Phe	649	483	19.12	14.49	TAT	Tyr	160	129	4.71	3.87
TTC	Phe	18	38	0.53	1.14	TAC	Tyr	14	24	0.41	0.72
TTA	Leu	459	377	13.52	11.31	TAA	*	0	0	0.00	0.00
TTG	Leu	73	107	2.15	3.21	TAG	*	0	0	0.00	0.00
CTT	Leu	26	46	0.77	1.38	CAT	His	46	45	1.36	1.35
CTC	Leu	2	1	0.06	0.03	CAC	His	0	2	0.00	0.06
CTA	Leu	12	33	0.35	0.99	CAA	Gln	30	30	0.88	0.90
CTG	Leu	0	8	0.00	0.24	CAG	Gln	8	26	0.24	0.78
ATT	Ile	260	188	7.66	5.64	AAT	Asn	146	99	4.30	2.97
ATC	Ile	6	13	0.18	0.39	AAC	Asn	8	5	0.24	0.15
ATA	Met	129	166	3.80	4.98	AAA	Lys	85	67	2.50	2.01
ATG	Met	17	19	0.50	0.57	AAG	Lys	14	33	0.41	0.99
GTT	Val	125	143	3.68	4.29	GAT	Asp	60	55	1.77	1.65
GTC	Val	2	8	0.06	0.24	GAC	Asp	6	5	0.18	0.15
GTA	Val	91	78	2.68	2.34	GAA	Glu	48	54	1.41	1.62
GTG	Val	22	19	0.65	0.57	GAG	Glu	26	35	0.77	1.05
TCT	Ser	85	90	2.50	2.70	TGT	Cys	33	41	0.97	1.23
TCC	Ser	1	13	0.03	0.39	TGC	Cys	1	0	0.03	0.00
TCA	Ser	29	38	0.85	1.14	TGA	Trp	50	59	1.47	1.77
TCG	Ser	3	7	0.09	0.21	TGG	Trp	12	28	0.35	0.84
CCT	Pro	49	41	1.44	1.23	CGT	Arg	25	16	0.74	0.48
CCC	Pro	1	4	0.03	0.12	CGC	Arg	0	1	0.00	0.03
CCA	Pro	16	28	0.47	0.84	CGA	Arg	3	13	0.09	0.39
CCG	Pro	3	4	0.09	0.12	CGG	Arg	3	3	0.09	0.09
ACT	Thr	67	62	1.97	1.86	AGT	Ser	89	86	2.62	2.58
ACC	Thr	0	13	0.00	0.39	AGC	Ser	4	5	0.12	0.15
ACA	Thr	18	30	0.53	0.90	AGA	Ser	103	95	3.03	2.85
ACG	Thr	2	4	0.06	0.12	AGG	Ser	20	35	0.59	1.05
GCT	Ala	61	53	1.80	1.59	GGT	Gly	77	101	2.27	3.03
GCC	Ala	0	7	0.00	0.21	GGC	Gly	3	8	0.09	0.24
GCA	Ala	14	20	0.41	0.60	GGA	Gly	51	58	1.50	1.74
GCG	Ala	5	4	0.15	0.12	GGG	Gly	24	31	0.71	0.93

### Transfer RNA and ribosomal RNA genes

Twenty-two discrete nucleotide sequences (ranging from 53 to 60 bp for *B. xylophilus* and 52 to 59 bp for *P. vulnus*) were predicted to fold into secondary structures of tRNAs (Additional files 1 and 2), similar to those found in other published nematode mtDNAs [32-35]. The predicted structures of tRNA genes in both species include an amino-acyl stem of seven nucleotide pairs (ntp), a DHU stem of 4 ntp with a loop, an anticodon stem of 5 ntp with a loop, and a TV replacement loop. Twenty of the 22 tRNA genes of both species have a unique feature in that the TΨC arm and variable loop are replaced by a TV replacement loop. The two exceptions are the serine tRNAs (*trnS1* and *trnS2*) that lack a DHU arm, but have a TΨC stem-loop structure. These features are found in other nematode mtDNAs [22-24,32-35], except for *T. spiralis* in which some tRNAs have the canonical cloverleaf structures [29]. The small-subunit ribosomal RNA (*rrnS*) and large-subunit ribosomal RNA (*rrnL*) of each mtDNA were initially identified by comparing with homologous gene sequence of other nematode species. The *rrnL* (948 bp) and *rrnS* (700 bp) for *B. xylophilus* are positioned between *trnH* and *nad3,* and between *trnE* and *trnS2,* respectively. The *rrnL* (895 bp) and *rrnS* (686 bp) for *P. vulnus* are positioned between *trnL1* and *nad3,* and between *cob* and *trnY*, respectively.

### Non-coding regions

For the *B. xylophilus* mtDNA, a total of 9 intergenic sequences, ranging from 1 to 1,650 bp, were found. Of these, the largest non-coding region (NCR; 1,650 bp), located between *nad4* and *cox1* genes, contains four identical repeat units of 147-nt, four identical repeat units of 101-nt, plus three identical repeat units of 56-nt with only a single nucleotide mismatch in the first repeat of the 56-nt repeat units. This NCR is extremely A+T-rich (98.7%), much higher than the entire mtDNA sequence (83.5%). In *P. vulnus* mtDNA, 24 intergenic sequences were found, ranging from a singleton to 6,847 bp (8,821 bp in total), accounting for 40.7% of the genome sequence. Among these, two non-coding regions (NCRs) are very conspicuous due to their lengths; NCR1 (6,847 bp) between *cox1* and *trnH*, and NCR2 (901 bp) between *trnH* and *trnL1* (Table 1). There are three repeated units of a 494-bp sequence (with only two between-unit nucleotide differences) found in the 5’ region of NCR1. The mitochondrial NCR in many metazoans contains a sequence motif for replication origin (the control region) that varies from a few hundred bp to tens of kb, with tandemly repeated sequence blocks in a ‘head-to-tail’ fashion. The presence of lengthy repeated segments in non-coding regions is responsible for exceptionally large mtDNA genome size and often contributes to genome size variation among and within individuals (i.e., size-variant heteroplasmy) and/or species in some other metazoans including American shad [36], bark weevils [37], root-knot nematodes [27], scallops [38] and some mermithid nematodes [28,39]. Comparative analysis of size variants might be useful for estimating the genetic structure of populations within species and between closely related species [40].

### Mitochondrial phylogeny among major chromadorean groups

**Figure 2:**
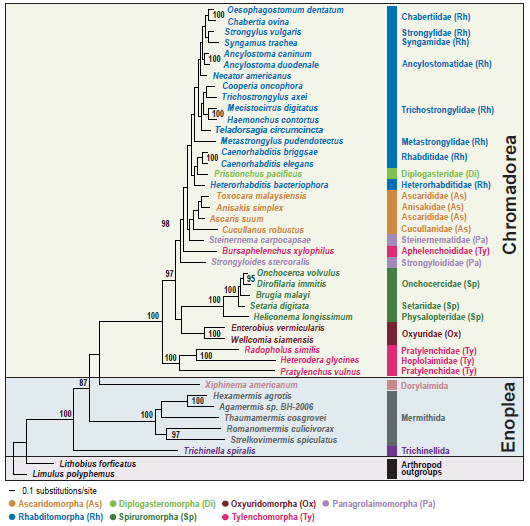
**Single maximum likelihood tree with values from the separate bootstrap analysis shown at internal nodes when 70% or greater.** Analysis of nucleotide sequences for 12 protein-coding genes with third codon positions included (11,784 characters) for 41 nematode mitochondrial genomes inferred using RAxML (see methods for analysis details). The classification of De Ley and Blaxter [12,13] is used to label each tree figure.

**Figure 3:**
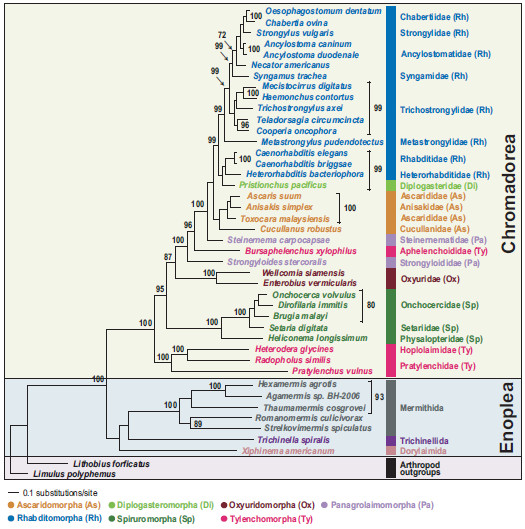
**Single maximum likelihood tree with values from the separate bootstrap analysis shown at internal nodes when 70% or greater.** Analysis of amino acid sequences (3,928 characters) for 12 protein-coding genes was conducted using RAxML.

**Figure 4:**
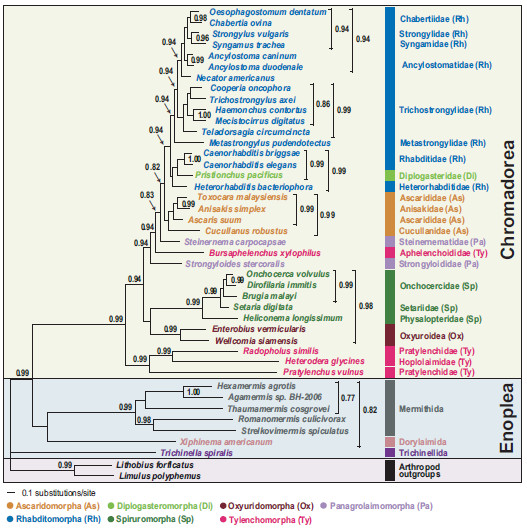
**Phylogenetic tree from Bayesian analysis of nucleotide sequences for 12 protein-coding genes with third codon positions included (11,784 characters).** The best-fit substitution model for each of 12 genes was estimated using the AIC criterion implemented in MrModeltest 2.3. The resulting best-fit model for each of 12 genes was then used for Bayesian analysis. Bayesian posterior probability values (BPP), shown above the nodes, were estimated after the initial 200 trees (the first 2x10^5^ generations) was discarded as burn-in (see Methods for analysis details).

**Figure 5:**
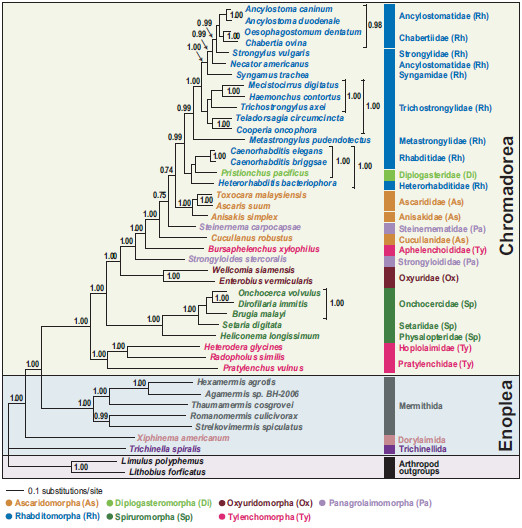
**Phylogenetic tree from Bayesian analysis of amino sequences for 12 protein-coding genes for 41 nematode mitochondrial genomes.** Bayesian posterior probability values (BPP), shown above the nodes, were estimated after the initial 200 trees (the first 2×10^5^ generations) were discarded as burn-in (see methods for analysis details).

We conducted Bayesian and maximum likelihood phylogenetic analyses of nucleotide (NT) and amino acid (AA) sequence datasets (12 protein-coding genes, 11,784 NT characters, 3,928 AA characters) for 41 nematode species, including the two newly sequenced species*.* The sequence alignments are deposited in TreeBASE (http://www.treebase.org [submission ID number: 13610 for the NT and 13609 for the AA]). Phylogenetic relationships were mainly consistent with previous reports based on mitochondrial genome analysis [22-24,35]. Chromadoreans formed a monophyletic group in all analyses with robust nodal support (100% bootstrap percentage [BP] in ML analyses (Figures 2 and 3) and 0.99-1.0 Bayesian posterior probability [BPP], (Figures 4 and 5). Within the Chromadorea ‘clade III’ (Ascaridomorpha, Oxyuridomorpha, and Spiruromorpha) as previously recovered from SSU rDNA [7] was not monophyletic, regardless of the dataset analyzed or tree-building method. The position of Oxyuridomorpha within Chromadorea varies among the mtDNA analyses. For the NT dataset, Oxyuridomorpha was sister to the Spiruromorpha (Figures 2 and 4) with high nodal support (89% BP in ML and 0.98 BPP in BI), but in the AA dataset, Oxyuridomorpha was sister to a large chromadorean clade (excluding Spiruromorpha and Tylenchoidea), with moderate (87% BP in ML) or strong (1.00 BPP) support (Figures 3 and 5). The relationship of *Pristionchus pacificus*, the only representative of the free-living infraorder Diplogasteromorpha, differed among analyses. For the NT dataset, *P. pacificus* was nested within certain Rhabditomorpha (sampled Rhabditoidea, i.e., *C. elegans, C. briggsae,* and *H. bacteriophora*) and this relationship was strongly supported (97% BP in ML (Figure 2) and 0.99 BPP in BI (Figure 4). For the AA dataset, the position of *P. pacificus* differed by inference method, with ML (Figure 3) showing *P. pacificus* as sister to the Rhabditoidea clade. In contrast, BI of the AA data (Figure 5) depicted *P. pacificus* as nested within the sampled Rhabditoidea, with strong support (1.00 BPP).

**Figure 6:**
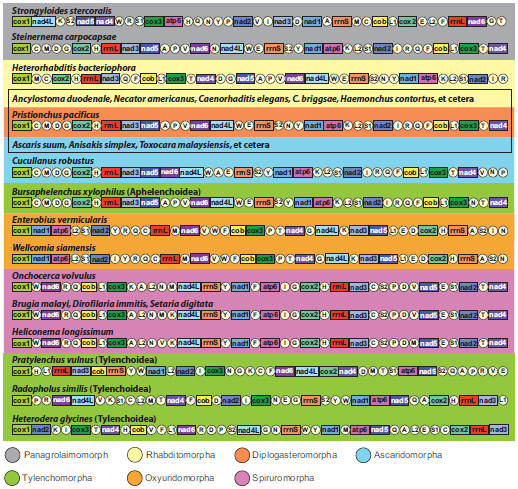
**Linearized representation of the mitochondrial gene arrangement for representatives of major chromadorean nematode clades, including two newly determined species (*B. xylophilus *and *P. vulnus*) in this study.** Gene and genome size are not to scale. All genes are transcribed in the same direction (from left to right). The tRNAs are designated by single-letter abbreviation and two leucine and two serine tRNA genes are labeled, according to their anticodon sequence, as L1 (*trnL-uag*), L2 (*trnL-uaa*), S1 (*trnS-ucu*), and S2 (*trnS-uga*), respectively. The AT-rich non-coding region (NCR) is not indicated. The rectangular box indicates the most common gene arrangement pattern shared among the majority of Rhabditomorpha-Ascaridomorpha members.

Gene order pattern in metazoan mitochondrial DNA has often been used as an additional tool for inferring relationships [41-43]. There are some reports of lineage-specific idiosyncratic gene order patterns in metazoans including nematodes (e.g., enoplean nematodes [39], mollusks [44,45], tunicates [46], crustacean arthropods [47]), and these cases illustrate that phylogenetic interpretation of gene arrangement data should be made with great caution. For example, gene order patterns for Enoplean species are not only very diverse, but radical gene rearrangements occur between relatively closely related species [39]. In contrast, mitochondrial gene order of chromadoreans is more conserved with substantial gene order pattern similarity (in most cases only translocations of tRNAs) within groups inferred to be monophyletic based on analysis of mtDNA sequences. With the exception of *S. sterocoralis* and *H. bacteriophora* in which substantial idiosyncratic gene arrangements distinguish them from other ordinal members (Figure 6), shared gene order patterns for chromadorean species has been interpreted as an additional indicator of their phylogenetic affinity [22-24]. The highly similar gene arrangement shared among most Rhabditomorpha, Ascaridomorpha, and the few representatives of Diplogasteromorpha and Aphelenchoidea for which data are available, may reflect common ancestry. However, within this clade, gene order data provides little information on relationships due to the high overall similarity.

### Phylogenetic relationships of Aphelenchoidea and Tylenchoidea

One aim of the present study was to assess Tylenchomorpha relationships, and specifically between *B. xylophilus* and species of Tylenchoidea. The hypothesis of Tylenchomorpha monophyly was examined with respect to relationships among chromadorean lineages. The phylogenetic trees inferred from both nucleotide and amino acid sequences are in agreement that Tylenchomorpha is not monophyletic (Figures 2, 3, 4, 5 and Additional files 3 and 4). Instead, *B. xylophilus* (Aphelenchoididae) was consistently found within the clade consisting of Rhabditomorpha, Panagrolaimomorpha, Diplogasteromorpha, and Ascaridomorpha (Figures 2, 3, 4 and 5), and usually (except Figure 5) “between” *Strongyloides stercoralis* and *Steinernema carpocapsae* (the sampled Panagrolaimomorpha). The clade of sampled Tylenchoidea, consisting of *Radopholus similis* (burrowing nematode), *Heterodera glycines* (soybean cyst nematode) and *Pratylenchus vulnus* (walnut root-lesion nematode), was sister to the other sampled Chromadorea in all analyses with very high nodal support (100% BP in ML (Figure 2) and 0.99 BPP in BI (Figure 4) for the NT dataset and 100% BP in ML (Figure 3) and 1.00 BPP in BI (Figure 5) for the AA dataset). This result indicates that there is no support from the mtDNA genome tree for a sister-group relationship between Tylenchoidea and *B. xylophilus* (Aphelenchoididae), or monophyly of Tylenchomorpha as currently defined [12,13]. Statistical evaluation of the alternative phylogenetic tree of highest likelihood with the constraint of Tylenchoidea plus *B. xylophilus* monophyly was a significantly worse interpretation of these mtDNA data (Table 4; Additional file 5). Phylogenetic hypotheses based on nuclear SSU rDNA also do not support monophyly of Tylenchida-Aphelenchoidea [7,9-11], but with different relationships recovered than for mtDNA. In SSU trees including the most compre-hensive sampling for Tylenchomorpha, aphelenchs are polyphyletic, with fungal-feeding taxa such as *Aphelenchus* spp. (Aphelenchidae) more closely related to plant-parasitic Tylenchoidea [7,9,11], and the Aphelenchoididae such as *B. xylophilus* and *Aphelenchoides* spp. either more closely related to Panagrolaimomorpha, or nested among a polyphyletic assemblage of panagrolaimomorph taxa [7,9-11]. Lack of appropriate taxon sampling for mtDNA genomes precludes direct comparison with these SSU results or statistical evaluation of certain alternative topologies, highlighting the need to sequence mtDNA genomes representing a greater diversity of Nematoda, especially additional Tylenchina (e.g., cephalobs, panagrolaims, aphelenchs *Aphelenchus* spp.]), and other families and superfamilies within Tylenchomorpha. This lack of taxon representation in the mtDNA genome tree may influence the sister-group relationship of Tylenchoidea to other Chromadorea, because adding taxa (new lineages) tends to break long branches, such as those characteristic of *P. vulnus, R. similis,* and *H. glycines*.

**Table 4:** **Results of Shimodaira-Hasegawa ML test (RAxML) for comparisons of alternative tree topologies to the best ML tree (Figure**2**) based on all nucleotide data (11,784 characters) and partitioned (by gene) analysis**

**Phylogenetic hypothesis**	**Difference in -lnL**	**S.D.**	**P value significant (0.01)**
Constrained monophyly			
1. Tylenchomorpha	93	30	yes
2. Tylenchoidea + Diplogasteromorpha	216	27	yes
3. Aphelenchoididae + Diplogasteromorpha	105	26	yes
4. Oxyuridomorpha + Tylenchoidea	54	15	yes

Alternative topologies tested are provided in Additional file 5.

In the mtDNA analyses of Tylenchoidea, the two sampled members of Pratylenchidae were not sister taxa, instead, *R. similis* (Pratylenchidae) was sister to *H. glycines* (Hoplolaimidae) with very strong support (e.g., 100% BP in ML (Figure 2) and 0.99 BPP in BI (Figure 4)) for the NT dataset. In terms of mtDNA gene arrangement patterns, *R. similis* and *H. glycines* share gene boundaries for *atp6-nad5-trnQ-trnA, rrnL-nad3, trnI-cox3, trnT-nad4,* and *trnS1-trnC*, whereas these two species share less gene order similarity with *P. vulnus* (Figure 6)*.* The monophyly of Tylenchoidea was strongly supported with *P. vulnus* (Pratylenchidae) sister to *R. similis* and *H. glycines* (100% BP in ML (Figure 2) and 0.99 BPP in BI (Figure 4) for the NT dataset and 100% BP in ML (Figure 3) and 1.00 BPP in BI (Figure 5) for the AA dataset, respectively). Comprehensive phylogenetic hypotheses based on SSU rDNA sequences also strongly support the monophyly of Tylenchomorpha, but not Pratylenchidae [11]. As in the mtDNA results, analysis of SSU rDNA shows that *R. similis* is more closely related to *Heterodera* spp. than to *P. vulnus* or other *Pratylenchus* spp. [11]. Thus, phylogenetic analysis of both SSU and mtDNA genome data independently show that Pratylenchidae, as traditionally defined, is not a natural group.

Thorne [48] first proposed that aphelenchs and tylenchs should be treated as two superfamilies (Aphelenchoidea and Tylenchoidea) within a separate order (Tylenchida), distinguishing this group from other nematodes. Subsequently, taxonomic authorities have offered several different interpretations for the relationships and taxonomic ranking of these nematodes. Many systematists have given special emphasis to morphological similarities shared between aphelenchs and tylenchs, as discerned by light microscopy, such as the protrusible stomatostylet, esophageal structures, and genital structure in females [15,16,18,49-53]. Maggenti [52] hypothesized that plant parasitism in nematodes evolved from fungal-feeding ancestors, and that the progenitors of these taxa were diplogasteromorph-like. Similarly, Poinar [54] emphasized a close relationship between tylenchs, aphelenchs and diplogasteromorphs based on similarities in pharyngeal structure. Later, Maggenti [18,50] recognized tylenchs and aphelenchs as separate orders within a subclass (Diplogasteria). This ‘diplogasteromorph origin’ was hypothesized on the basis of the general similarity of esophageal structure among tylenchs, aphelenchs, and diplogasteromorphs and inferences concerning development of stoma armature (stylets) from stomatal features of certain diplogasteromorphs. Siddiqi [14,17,19] argued that these morphologically similar stylets arose independently in tylenchs and aphelenchs, asserting that plant parasitism in these two groups had separate evolutionary origins, with the former evolved from cephalobomorph/oxyuridomorph-like ancestors and the latter from diplogasteromorph-like ancestors. Our mitochondrial genome phylogeny (Figures 2, 3, 4, 5 and Additional files 3 and 4) did not support a close relationship between diplogasteromorphs (represented by *P. pacificus*) and either tylenchs (*R. similis, H. glycines,* and *P. vulnus*) or aphelenchs (*B. xylophilus*). Similarly, oxyuridomorphs (*E. vermicularis* and *W. siamensis*) and tylenchs did not form a clade. Tree topology tests indicated that alternative phylogenies constraining a sister-group relationship between Diplogasteromorpha and Tylenchoidea, Diplogasteromorpha and Aphelenchoidea, or Oxyuridomorpha and Tylenchoidea were significantly worse interpretations of these mtDNA data (Table 4; Additional file 5). Considering the gene arrangement pattern among these nematodes (with the exception of *S. stercoralis*, *H. bacteriophora,* and *C. robustus*, which have idiosyncratic gene arrangements [24,35]) the sampled Rhabditomorpha, Panagrolaimomorpha, Diplogasteromorpha, Ascaridomorpha and *B. xylophilus* have almost identical patterns with only a translocation of *trnN* for *B. xylophilus* (Figure 6). Indeed, except for the non-coding region (NCR), 33 of the species within the clade containing *B. xylophilus* share this most common gene arrangement type. In contrast, the gene order of only four blocks comprising two consecutive genes (*nad6-nad4L, nad2-trnI, cox3-trnN,* and *rrnL-nad3*) is shared between *P. vulnus, R. similis* and *B. xylophilus*. Moreover, only a single block (*rrnL-nad3*) is shared between *B. xylophilus* and all three tylenchs (*P. vulnus, R. similis* and *H. glycines*).

## Conclusions

In this study, we investigated the phylogenetic relationships of chromadorean nematodes, including new sequences of Tylenchomorpha previously unrepresented in mtDNA genome trees. The mitochondrial genomes of *B. xylophilus* and *P. vulnus* are 14,778 bp and 21,656 bp, respectively, and identical to all other chromadorean nematode mtDNAs in that they contain 36 genes (lacking *atp8*) encoded in the same direction. *Pratylenchus vulnus* has the largest mitochondrial genome of any chromadorean nematode sequenced, due to a large fraction (54%) of non-coding sequence. Phylogenetic analyses of nucleotide and amino acid sequence datasets using maximum likelihood and Bayesian methods did not support monophyly of Tylenchomorpha. Instead, *B. xylophilus* was nested within the Rhabditomorpha+Ascaridomorpha+Panagrolaimomorpha+Diplogasteromorpha clade, and Tylenchoidea (represented by *P. vulnus, H. glycines*, and *R. similis*) was sister to all analyzed chromadoreans. Comparison of gene arrangement data was also consistent with the phylogenetic relationships as inferred from sequence data. Statistical comparison of alternative tree topologies revealed that constraining Tylenchomorpha to be monophyletic was a significantly worse interpretation of these mtDNA data. These results confirm previous findings based on nuclear SSU rDNA, indicating that aphelench and tylench plant parasites do not share an exclusive most recent common ancestor, and revealing that certain morphological similarities between these stylet-bearing nematodes must result from convergent evolution.

## Methods

### Sampling of specimens

Live nematodes of *B. xylophilus* were isolated from infected pine trees *Pinus densiflora* (Jinju, Gyeongnamdo Province, South Korea) and maintained in *Botrytis*-containing agar culture plates until they were used for total genomic DNA extraction. The root-lesion nematode *P. vulnus* was isolated in Davis, CA, U.S.A. and established and maintained on carrot disk culture in the laboratory. Specimens harvested from disk culture were used for the genomic DNA extraction.

### Molecular techniques

Total genomic DNA was extracted from pooled nematodes of each species using the Qiagen DNA extraction kit according to the manufacturer’s protocol. Initially, partial mtDNA fragments were amplified from different regions of mtDNA using universal primers or primer sets designed from the conserved regions of closely related nematode species. Four partial fragments from *cox1, rrnS, cob* and *cox2* gene regions were PCR-amplified for *B. xylophilus* using universal primer sets (LCO1490/HCO2198, 12SL1091/12SH1478 and CtybL14841/CtybH15149 for *cox1*, *rrnS* and *cob,* respectively) and the primer set (COX2-F/R) designed directly from conserved regions of *cox2* sequences for chromadorean nematode sequences (See Additional file 6 for primer sequences). For *P. vulnus*, two partial fragments (from *cob* and *cox2* gene regions) were amplified using universal primer set CtybL14841/CtybH15149 for *cob* and the primer set (COX2-F/R). PCR reactions for both species were carried out in a 20 μl reaction volume containing 10 units of *Taq* polymerase (Roche), 2.5 mM dNTP mixture, 2.5 mM MgCl_2_, and 20 pmole of each primer with the following amplification conditions: one cycle of the initial denaturation step at 94°C for 2 min, followed by 35 cycles of denaturation at 94°C for 1 min, primer annealing at 43-48°C for 30 s and elongation at 72°C for 1 min. A final extension was performed at 72°C for 10 min. The nucleotide sequences obtained from these gene fragments for each of the two species were then used to design species-specific primers for long PCR amplification. Four overlapping long PCR products (ranging from 1.8 kb to 4 kb; see Additional file 6) covering the entire *B. xylophilus* mtDNA were obtained using the *B. xylophilus*-specific long PCR primer sets. Similarly, two overlapping long PCR-amplified fragments (ranging from 6 kb and 15 kb) covering the entire mtDNA of *P. vulnus* were obtained using the *P. vulnus*-specific long PCR primer sets. The long-PCR amplification was performed using the Expanded Long Template PCR System (Roche, USA) under the following conditions: 1 cycle of initial denaturation (2 min at 93°C), 30 cycles of denaturation–primer annealing–elongation (15 s at 93°C, 30 s at 50–60°C, and 13 min at 68°C), and 1 cycle of the final extension (10 min at 68°C). The long PCR products were gel-isolated and ligated using the TOPO XL cloning kit (Invitrogen), as recommended by the manufacturer. Cycle sequencing reactions for each of the cloned PCR products were performed in both directions using a “primer walking” strategy, and the sequences of overlapping fragments were double-checked and assembled to obtain the complete sequence of each mitochondrial genome.

### Gene annotation and phylogenetic analyses

Nucleotide sequences were initially analyzed using MEGA4. Each of the twelve mitochondrial protein-coding genes for *B. xylophilus* and *P. vulnus* was identified by recognizing the open reading frames and comparing the inferred amino acid sequences with those of other nematode species. The start and termination codons were predicted for 12 protein-coding genes by comparison of the amino acid sequences to homologous genes of other Chromadorean nematodes. Ribosomal RNA (rRNA) genes were also identified by comparison with other complete mitochondrial rRNAs of nematodes. The 22 tRNA genes for each mtDNA sequence were identified using the tRNAscan-SE program [55] or by manually inspecting potential secondary structures and anticodon sequences.

For phylogenetic analysis, two types of sequence datasets (nucleotide and amino acid sequences) for the 12 protein-coding genes of the complete mtDNA were obtained from 41 nematode species including *B. xylophilus* and *P. vulnus* (Additional file 7). Sequence data from two arthropods (*Lithobius forficatus* and *Limulus polyphemus*) were also included in the analyses as outgroups. Prior to multiple sequence alignment, nucleotide sequences of the 12 protein-coding genes were translated to amino acid sequences using the invertebrate mitochondrial genetic code. ClustalX with default options [56] was then used to perform multiple alignments of the amino acid sequences. The corresponding nucleotide sequences for these protein-coding genes were aligned based on indels inferred from the protein alignment; the web-based tool RevTrans [57], was used to place the indels in the nucleotide sequences. Phylogenetic analyses of both concatenated nucleotide and amino acid sequence datasets for all 12 protein-coding genes were performed using different tree-building methods (maximum likelihood [ML] and Bayesian inference [BI]). Maximum likelihood (ML) analysis was performed using RAxML 7.0.3 [58] and bootstrap ML analysis was performed using the rapid bootstrap resampling method of RAxML with 1000 replicates, using the CIPRES Science Gateway [59]. The likelihood scores of the competing hypotheses (the best ML tree versus alternative hypothesis) were compared using the Shimodaira-Hasegawa (S-H) test [60]. For both ML and BI, datasets were partitioned by gene for analysis. For BI, MrModeltest 2.3 (nucleotide dataset [61]) and ProtTest 2.4 (amino acid dataset [62]) programs were used to determine the best-fit model for each gene prior to analysis, respectively. Using the CIPRES Gateway, two runs of MrBayes were executed using four MCMC chains and 10^6^ generations, sampled every 1,000 generations. Each of the 12 genes was treated as a separate unlinked data partition. Bayesian posterior probability (BPP) values were determined after discarding the initial 200 trees (the first 2×10^5^ generations) as burn-in. In addition, ML and BI inference methods were used to analyze the nucleotide data, but with 3^rd^ positions of codons excluded (7,856 characters).

## Abbreviations

AA: Amino acid; *atp6* and *atp8*: Genes for ATP synthase subunits 6 and 8; BI: Bayesian inference; bp: Base pair; BP: Bootstrap percentage; BPP: Bayesian posterior probability; *cob*: Gene for cytochrome oxidase *b*; *cox1*–*cox3*: Genes for cytochrome oxidase *c* subunit 1–3; DHU: Dihydrouridine; dNTP: Deoxyribonucleotide triphosphate; kb: Kilo base; LSU: Large subunit nuclear ribosomal DNA; ML: Maximum likelihood; mtDNA: Mitochondrial DNA; *nad1–6,* and *nad4L*: Genes for NADH dehydrogenase subunits 1–6 and 4L; NCR: Non-coding region; nt: Nucleotide; NT: Nucleotide; PCR: Polymerase chain reaction; *rrnS* and *rrnL*: Genes for small and large mitochondrial ribosomal RNA subunits; SSU: Small subunit nuclear ribosomal DNA; tRNA: Transfer RNA.

## Competing interests

The authors have no competing interests to declare.

## Authors’ contributions

TS, JK, SHL, HH, SK, and GSM participated in laboratory work for mitochondrial genome sequencing. JKP designed the study, performed phylogenetic analyses, interpreted the results and drafted the manuscript. SAN also participated in phylogenetic analyses, interpretation of trees, and drafting the manuscript. All authors read and approved the final manuscript.

## Supplementary Material

Additional file 1 **The predicted secondary structures of 22 tRNAs for *Bursaphelenchus xylophilus. *** Click here for file

Additional file 2 **The predicted secondary structures of 22 tRNAs for *Pratylenchus vulnus.*** Click here for file

Additional file 3 **Single maximum likelihood tree with values from the separate bootstrap analysis shown at internal nodes when 70% or greater.** Analysis of nucleotide sequences for 12 protein-coding genes with third codon positions excluded (7,856 characters) for 41 nematode mitochondrial genomes inferred using RAxML (see methods for analysis details). Click here for file

Additional file 4 **Phylogenetic tree from Bayesian analysis of nucleotide sequences for 12 protein-coding genes with third codon positions excluded (7,856 characters).** The best-fit substitution model for each of 12 genes was estimated using the AIC criterion implemented in MrModeltest 2.3. The resulting best-fit model for each of 12 genes was then used for Bayesian analysis. Bayesian posterior probability values (BPP), shown above the nodes, were estimated after the initial 200 trees (the first 2x10^5^ generations) was discarded as burn-in (see methods for analysis details). Click here for file

Additional file 5 **Nexus file descriptions for alternative topologies tested for results reported in Table **4. Click here for file

Additional file 6 PCR primer information used in this study. Click here for file

Additional file 7 The species, taxonomy, and GenBank accession numbers for nematode species used in phylogenetic analyses in this study. Click here for file
