# Supplementary material for: Comparative analysis of complete mitochondrial genome sequences confirms independent origins of plant-parasitic nematodes
Source: BMC Evol Biol. 2013 Jan 18;13:12. doi: 10.1186/1471-2148-13-12 (PMC3558337; doi:10.1186/1471-2148-13-12)

Additional file 1

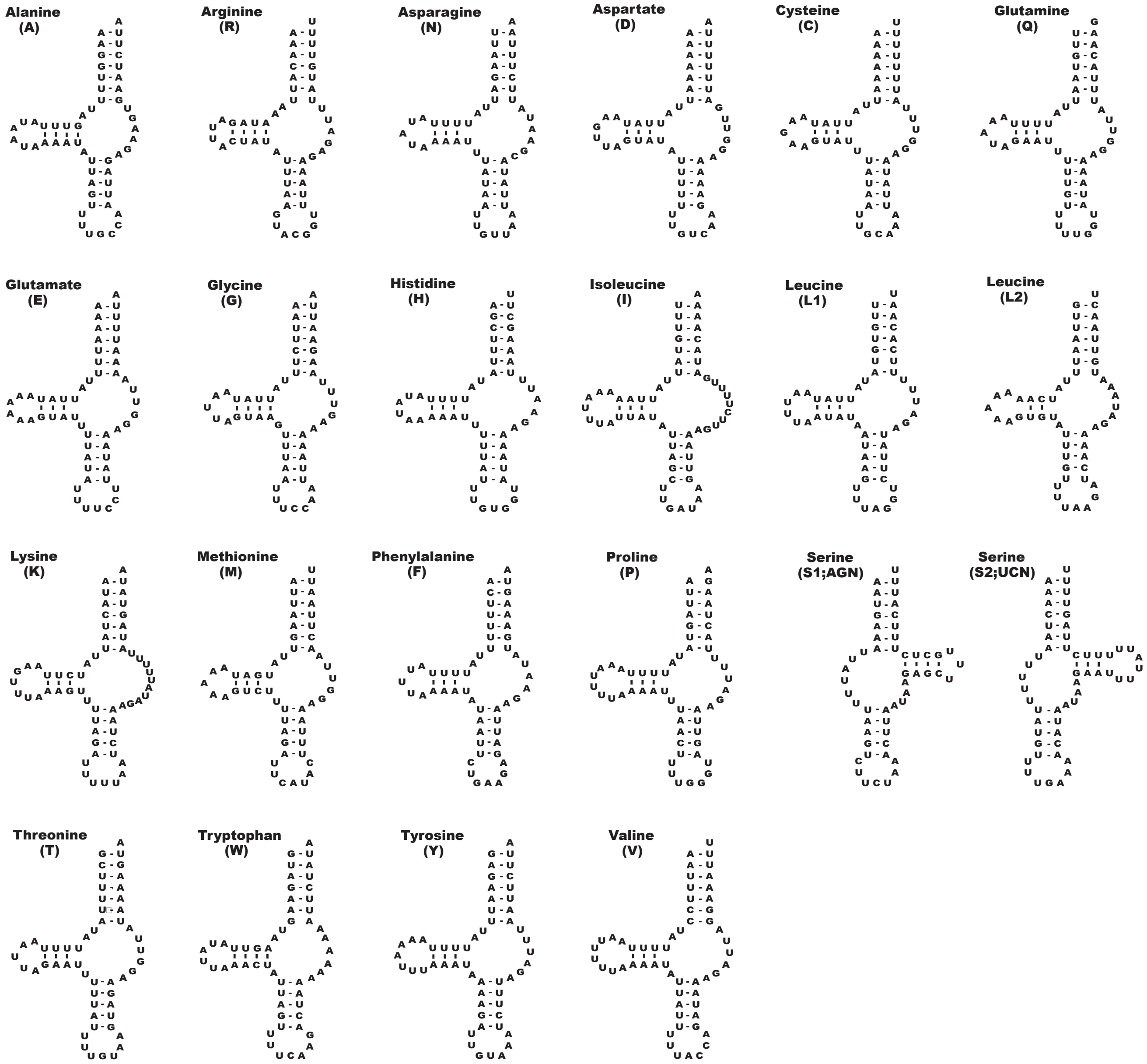

Supplement: Additional file 1 — The predicted secondary structures of 22 tRNAs for Bursaphelenchus xylophilus. [file 1471-2148-13-12-S1.pdf]
