# Supplementary material for: Comparative analysis of complete mitochondrial genome sequences confirms independent origins of plant-parasitic nematodes
Source: BMC Evol Biol. 2013 Jan 18;13:12. doi: 10.1186/1471-2148-13-12 (PMC3558337; doi:10.1186/1471-2148-13-12)

Additional file 3

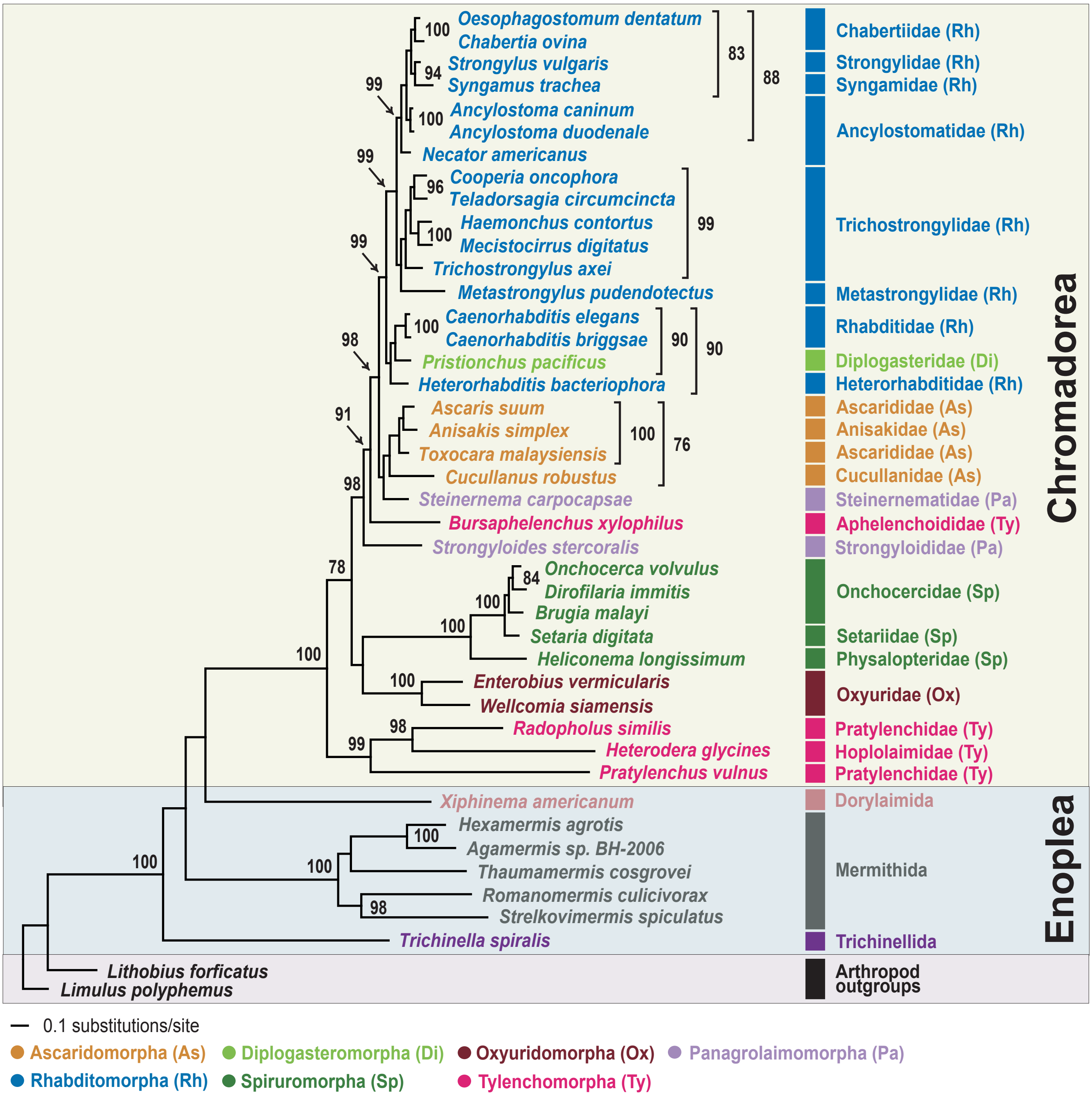

Supplement: Additional file 3 — Single maximum likelihood tree with values from the separate bootstrap analysis shown at internal nodes when 70% or greater. Analysis of nucleotide sequences for 12 protein-coding genes with third codon positions excluded (7,856 characters) for 41 nematode mitochondrial genomes inferred using RAxML (see methods for analysis details). [file 1471-2148-13-12-S3.pdf]
