# Supplementary material for: Comparative analysis of complete mitochondrial genome sequences confirms independent origins of plant-parasitic nematodes
Source: BMC Evol Biol. 2013 Jan 18;13:12. doi: 10.1186/1471-2148-13-12 (PMC3558337; doi:10.1186/1471-2148-13-12)

Additional file 4

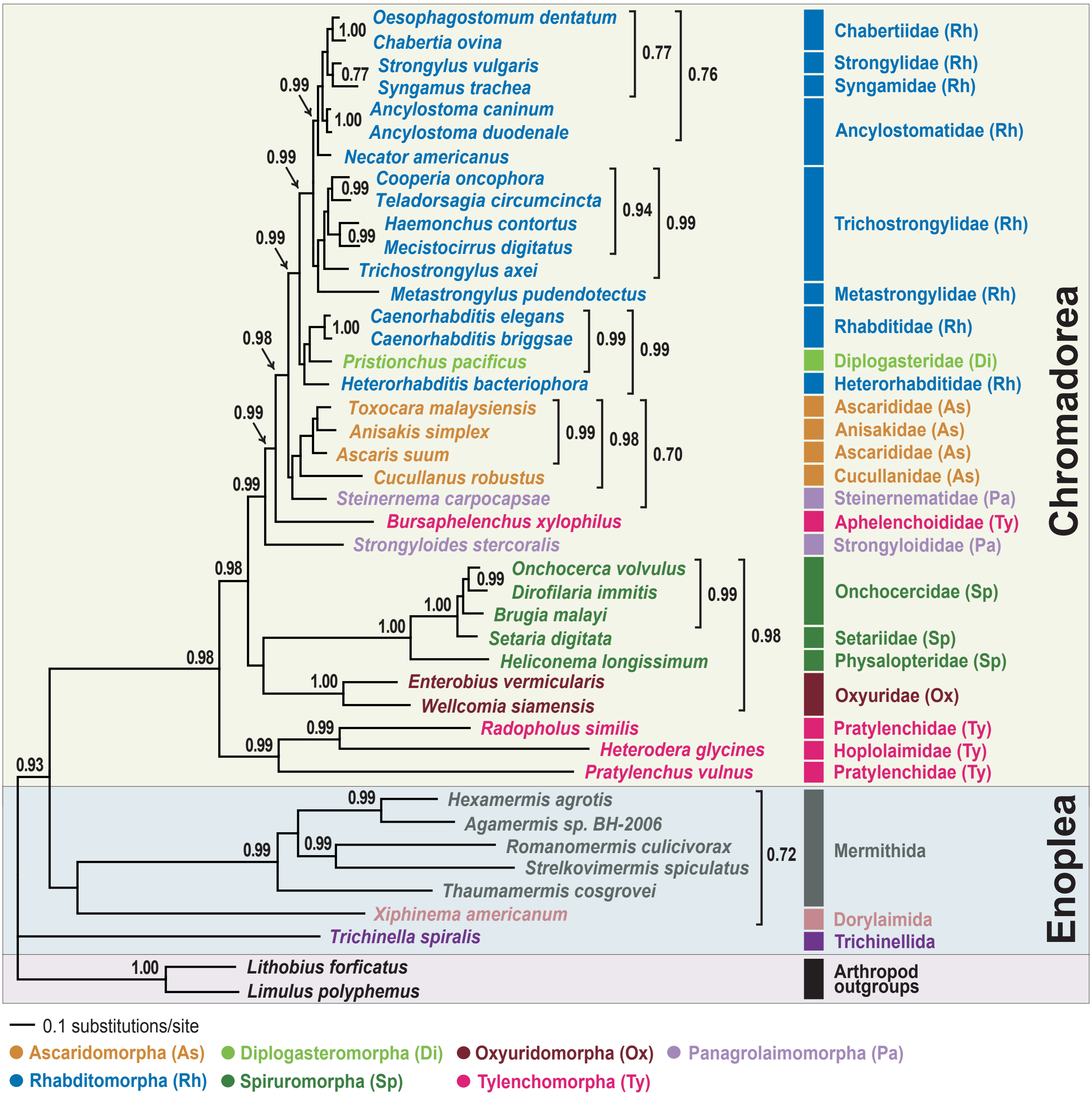

Supplement: Additional file 4 — Phylogenetic tree from Bayesian analysis of nucleotide sequences for 12 protein-coding genes with third codon positions excluded (7,856 characters). The best-fit substitution model for each of 12 genes was estimated using the AIC criterion implemented in MrModeltest 2.3. The resulting best-fit model for each of 12 genes was then used for Bayesian analysis. Bayesian posterior probability values (BPP), shown above the nodes, were estimated after the initial 200 trees (the first 2x10^5^ generations) was discarded as burn-in (see methods for analysis details). [file 1471-2148-13-12-S4.pdf]
