# Supplementary material for: Comparative analysis of complete mitochondrial genome sequences confirms independent origins of plant-parasitic nematodes
Source: BMC Evol Biol. 2013 Jan 18;13:12. doi: 10.1186/1471-2148-13-12 (PMC3558337; doi:10.1186/1471-2148-13-12)
Supplement: Additional file 5 — Nexus file descriptions for alternative topologies tested for results reported in Table 4. [file 1471-2148-13-12-S5.docx]

**Additional file 5. Nexus file descriptions for alternative topologies tested for results reported in Table 4.**

AlternateTREE1 = ((21,20),(41,(((((1,16),38),(30,33)),43),(((28,(29,18)),7),(((14,42),(19,(31,(6,(26,13))))),(34,(32,((((4,39),5),12),((((8,9),27),17),(23,((37,((15,22),(11,40))),((((10,25),(35,36)),(2,3)),24))))))))))));

AlternateTREE2 = (21,(20,(41,((((1,16),38),(30,33)),(43,(((14,42),(19,(31,(6,(26,13))))),(34,(7,(((((4,39),5),12),32),((((8,9),((28,(29,18)),27)),17),(23,((37,((15,22),(11,40))),((((10,25),(35,36)),(2,3)),24)))))))))))));

AlternateTREE3 = (21,(20,(41,((((1,16),38),(30,33)),(43,((28,(29,18)),(((14,42),(19,(31,(6,(26,13))))),(34,(((((4,39),5),12),32),((((8,9),(7,27)),17),(23,((37,((15,22),(11,40))),((((10,25),(35,36)),(2,3)),24)))))))))))));

AlternateTREE4 = (21,(20,(41,((((1,16),38),(30,33)),(43,(((28,(29,18)),(14,42)),((19,(31,(6,(26,13)))),(34,(7,(((((4,39),5),12),32),((((8,9),27),17),(23,((37,((15,22),(11,40))),((((10,25),(35,36)),(2,3)),24))))))))))))));

Taxon Translation=1Ag_BH20, 2An_cani, 3An_duod, 4An_simp, 5As_suum, 6Br_mala, 7Bu_xylo, 8Ca_brig, 9Ca_eleg, 10Ch_ovin, 11Co_onco, 12Cu_robu, 13Di_immi, 14En_verm, 15Ha_cont, 16He_agro, 17He_bact, 18He_glyc, 19He_long, 20Li_forf, 21Li_poly, 22Me_digi, 23Me_pude, 24Ne_amer, 25Oe_dent, 26On_volv, 27Pr_paci, 28Pr_vuln, 29Ra_simi, 30Ro_culi, 31Se_digi, 32St_carp, 33St_spic, 34St_ster, 35St_vulg, 36Sy_trac, 37Te_circ, 38Th_cosg, 39To_mala, 40Tr_axei, 41Tr_spir, 42We_siam, 43Xi_amer

**Taxon abbreviations**

1. Ag_BH20: *Agamermis sp.* BH-2006; 2. An_cani: *Ancylostoma caninum*; 3. An_duod: *Ancylostoma* *duodenale*; 4. An_simp: *Anisakis simplex*; 5. As_suum: *Ascaris suum*; 6. Br_mala: *Brugia malayi*; 7. Bu_xylo: *Bursaphelenchus xylophilus*; 8. Ca_brig: *Caenorhabditis briggsae*; 9. Ca_eleg: *Caenorhabditis elegans*; 10. Ch_ovin: *Chabertia ovina*; 11. Co_onco: *Cooperia oncophora*; 12. Cu_robu: *Cucullanus robustus*; 13. Di_immi: *Dirofilaria immitis*; 14. En_verm: *Enterobius vermicularis*; 15. Ha_cont: *Haemonchus contortus*; 16. He_agro: *Hexamermis agrotis*; 17. He_bact: *Heterorhabditis bacteriophora*; 18. He_glyc: *Heterodera glycines*; 19. He_long: *Heliconema longisimum*; 20. Li_forf: *Lithobius forficatus*; 21. Li_poly: *Limulus polyphemus*; 22. Me_digi: *Mecistocirrus digitatus*; 23. Me_pude: *Metastrongylus pudendotectus*; 24. Ne_amer: *Necator americanus*; 25. Oe_dent: *Oesophagostomum dentatum*; 26. On_volv: *Onchocerca volvulus*; 27. Pr_paci: *Pristionchus pacificus*; 28. Pr_vuln: *Pratylenchus vulnus*; 29. Ra_simi: *Radopholus similis*; 30. Ro_culi: *Romanomermis culicivorax*; 31. Se_digi: *Setaria digitata*; 32. St_carp: *Steinernema carpocapsae*; 33. St_spic: *Strelkovimermis spiculatus*; 34. St_ster: *Strongyloides stercoralis*; 35. St_vulg: *Strongylus vulgaris*; 36. Sy_trac: *Syngamus trachea*; 37. Te_circ: *Teladorsagia circumcincta*; 38. Th_cosg: *Thaumamermis cosgrovei*; 39. To_mala: *Toxocara malaysiensis*; 40. Tr_axei: *Trichostrongylus axei*; 41. Tr_spir: *Trichinella spiralis*; 42. We_siam: *Wellcomia siamensis*; 43. Xi_amer: *Xiphinema americanum*
