# Supplementary material for: Comparative analysis of complete mitochondrial genome sequences confirms independent origins of plant-parasitic nematodes
Source: BMC Evol Biol. 2013 Jan 18;13:12. doi: 10.1186/1471-2148-13-12 (PMC3558337; doi:10.1186/1471-2148-13-12)
Supplement: Additional file 6 — PCR primer information used in this study. [file 1471-2148-13-12-S6.docx]

**Additional file 6. PCR primer information used in this study**

| Primers | Sequence (5′–3′) | Source | Target species | Estimated size of PCR product |
| --- | --- | --- | --- | --- |
| LCO1490  LCO2198 | GGTCAACAACTCATAAAGATATTG  TAAACTTCAGGGTGACCAAAAAATCA | Folmer et al (1994)^1^ | *Bursaphelenchus xylophilus* | 700 bp |
| 12SL1091  12SH1478 | AAACTGGGATTAGATACCCCACTA  GAGGGTGACGGGGGGTGTGT | Kocher et al. 1989^2^ | *Bursaphelenchus xylophilus* | 450 bp |
| CtybL14841  CtybH15149 | AAAAAGCTTCCATCCAACATCTCAGCATGATAAA  AAACTGCAGCCCCTCAGAATGATATTTGTCCTCA | Kocher et al. 1989^2^ | *Bursaphelenchus xylophiluss* and *Pratylenchus vulnus* | 500 bp |
| COX2-F  COX2-R | GGACATCAGTGATATTGAAGATATG  GCTACCTTAATGTCCTCACGCTAAG | This study | *Bursaphelenchus xylophilus* and *Pratylenchus vulnus* | 700 bp |
| Bx-COX1-F  Bx-COX2-R | GWACWTTAGGKCATCCWGGTGGAAGWGTTG  GTTCAAGCCTGAATAACATCAGCAGAAG | This study | *Bursaphelenchus xylophilus* | 1.8 kb |
| Bx-COX2-F  Bx-12S-R | GGAGTTTWTTATGGTCAATGTTCG  GAGGACTCTCAAATTAAAACTCAACCTCC | This study | *Bursaphelenchus xylophilus* | 4 kb |
| Bx-12S-F  Bx-CytB-R | GGAGGTTGAGTTTTAATTTGAGAGTCCTC  GTAGCACCAGAAACACTAAAACCACTTC | This study | *Bursaphelenchus xylophilus* | 3.5 Kb |
| Bx-CytB-F  Bx-COX1-R | GAAGTGGTTTTAGTGTTTCTGGTGCTAC  GTTCAWCTAGTTCCWGCACCAGTATCAAC | This study | *Bursaphelenchus xylophilus* | 3.5 Kb |
| Pv-COX2-F  Pv-CytB-R | CATAGATTTATACCTATTGTAGTAGA  ATTGGTTCTTAAACCAAAACCTCCCC | This study | *Pratylenchus vulnus* | 6 Kb |
| Pv-CytB-F  Pv-COX2-R | CCCTTAGAGCTAGGTGATCCTTTAGG  GGAACTACACATCGATTATCTACATC | This study | *Pratylenchus vulnus* | 15 Kb |

The IUPAC codes were used for W (T, A), R (A, G) and Y (C, T)

^1^Folmer O, Black M, Hoeh W, Lutz R, Vrijenhoek R: **DNA primers for amplification of mitochondrial cytochrome *c* oxidase subunit I from diverse metazoan invertebrates.** *Mol Mar Biol Biotech* 1994, **3:**294-299.

^2^Kocher TD, Thomas WK, Meyer A, Edwards SVS, Villa Blanca FX, Wilson AC: **Dynamics of mitochondrial DNA evolution in animals: amplification and sequencing with conserved primers.** *Proc Natl Acad Sci USA* 1989, **86:**6196-6200.
