# Supplementary material for: Comparative analysis of complete mitochondrial genome sequences confirms independent origins of plant-parasitic nematodes
Source: BMC Evol Biol. 2013 Jan 18;13:12. doi: 10.1186/1471-2148-13-12 (PMC3558337; doi:10.1186/1471-2148-13-12)
Supplement: Additional file 7 — The species, taxonomy, and GenBank accession numbers for nematode species used in phylogenetic analyses in this study. [file 1471-2148-13-12-S7.docx]

**Additional file 7.** The species, taxonomy, and GenBank accession numbers for nematode species used in phylogenetic analyses in this study. Classification according to De Ley and Blaxter [12,13].

| **Species** | **Classification** | **GenBank accession number** |
| --- | --- | --- |
| *Agamermis sp*. BH-2006 | Mermithida (Mermithidae)^1^ | NC_008231 |
| *Ancylostoma caninum* | Rhabditomorpha (Ancylostomatidae)^2^ | NC_012309 |
| *Ancylostoma duodenale* | Rhabditomorpha (Ancylostomatidae)^2^ | NC_003415 |
| *Anisakis simplex* | Ascaridomorpha (Anisakidae)^2^ | NC_007934 |
| *Ascaris suum* | Ascaridomorpha (Ascarididae)^2^ | NC_001327 |
| *Brugia malayi* | Spiruromorpha (Onchocercidae)^2^ | NC_004298 |
| *Bursaphelenchus xylophilus* | Tylenchomorpha (Aphelenchoididae)^2^ | GQ_332424 |
| *Caenorhabditis briggsae* | Rhabditomorpha (Rhabditidae)^2^ | NC_009885 |
| *Caenorhabditis elegans* | Rhabditomorpha (Rhabditidae)^2^ | NC_001328 |
| *Chabertia ovina* | Rhabditomorpha (Chabertiidae)^2^ | NC_013831 |
| *Cooperia oncophora* | Rhabditomorpha (Trichostrongylidae)^2^ | NC_004806 |
| *Cucullanus robustus* | Ascaridomorpha (Cucullanidae)^2^ | GQ_332426 |
| *Dirofilaria immitis* | Spiruromorpha (Onchocercidae)^2^ | NC_005305 |
| *Enterobius vermicularis* | Oxyuridomorpha (Oxyuridae)^2^ | NC_011300 |
| *Haemonchus contortus* | Rhabditomorpha (Trichostrongylidae)^2^ | NC_010383 |
| *Heliconema longissimum* | Spiruromorpha (Physalopteridae)^2^ | GQ_332423 |
| *Heterodera glycines* | Tylenchomorpha (Hoplolaimidae)^2^ | HM_640930 |
| *Heterorhabditis bacteriophora* | Rhabditomorpha (Heterorhabditidae)^2^ | NC_008534 |
| *Hexamermis agrotis* | Mermithida (Mermithidae)^1^ | NC_008828 |
| *Necator americanus* | Rhabditomorpha (Ancylostomatidae)^2^ | NC_003416 |
| *Mecistocirrus digitatus* | Rhabditomorpha (Trichostrongylidae)^2^ | NC_013848 |
| *Metastrongylus pudendotectus* | Rhabditomorpha (Metastrongylidae)^2^ | NC_013813 |
| *Oesophagostomum dentatum* | Rhabditomorpha (Chabertiidae)^2^ | NC_013817 |
| *Onchocerca volvulus* | Spiruromorpha (Onchocercidae)^2^ | NC_001861 |
| *Pratylenchus vulnus* | Tylenchomorpha (Pratylenchidae)^2^ | GQ_332425 |
| *Pristionchus pacificus* | Diplogasteromorpha (Diplogasteridae)^2^ | NC_015245 |
| *Radopholus similis* | Tylenchomorpha (Pratylenchidae)^2^ | NC_013253 |
| *Romanomermis culicivorax* | Mermithida (Mermithidae)^1^ | NC_008640 |
| *Setaria digitata* | Spiruromorpha (Setariidae)^2^ | NC_014282 |
| *Steinernema carpocapsae* | Panagrolaimomorpha (Steinernematidae)^2^ | NC_005941 |
| *Strelkovimermis spiculatus* | Mermithida (Mermithidae)^1^ | NC_008047 |
| *Strongylus vulgaris* | Rhabditomorpha (Strongylidae)^2^ | NC_013818 |
| *Strongyloides stercoralis* | Panagrolaimomorpha (Strongyloididae)^2^ | NC_005143 |
| *Syngamus trachea* | Rhabditomorpha (Syngamidae)^2^ | NC_013821 |
| *Teladorsagia circumcincta* | Rhabditomorpha (Trichostrongylidae)^2^ | NC_013827 |
| *Thaumamermis cosgrovei* | Mermithida (Mermithidae)^1^ | NC_008046 |
| *Toxocara malaysiensis* | Ascaridomorpha (Ascarididae)^2^ | NC_010527 |
| *Trichinella spiralis* | Trichinellida (Trichinellidae)^1^ | NC_002681 |
| *Trichostrongylus axei* | Rhabditomorpha (Trichostrongylidae )^2^ | NC_013824 |
| *Wellcomia siamensis* | Oxyuridomorpha (Oxyuridae)^2^ | GQ_332427 |
| *Xiphinema americanum* | Dorylaimida (Longidoridae)^1^ | NC_005928 |
| *Lithobius forficatus* | Myriapoda, Arthropoda | NC_002629 |
| *Limulus polyphemus* | Chelicerata, Arthropoda | NC_003057 |

^1^ Class Enoplea

^2^ Class Chromadorea
